# The Remote Intradural Migration of Polyethylene Glycol-Based Hydrogel Sealant Following Lumbar Laminectomy: A Case Report

**DOI:** 10.3390/jcm14051472

**Published:** 2025-02-22

**Authors:** Barnabas Obeng-Gyasi, Trenton A. Line, Whitney Brown, Anoop S. Chinthala, Nathan J. Kussow, Gordon Mao

**Affiliations:** Department of Neurological Surgery, Indiana University School of Medicine, Indianapolis, IN 46202, USA; bobenggy@iu.edu (B.O.-G.); treline@iu.edu (T.A.L.); whabrown@iu.edu (W.B.); aschinth@iu.edu (A.S.C.); nkussow@iu.edu (N.J.K.)

**Keywords:** dural sealant, cerebrospinal fluid leak, spinal surgery, surgical complications, polyethylene glycol, postoperative complications

## Abstract

**Background/Objectives**: Synthetic polyethylene glycol (PEG)-based hydrogel sealants, such as Adherus, are commonly used in spinal surgeries to achieve watertight dural closure and prevent cerebrospinal fluid (CSF) leaks. This case report describes an unusual instance of suspected hydrogel sealant migration resulting in an intradural collection at a spinal level remote from the original surgery. **Methods**: A 57-year-old female with a history of osteoarthritis and prediabetes underwent a minimally invasive L5-S1 laminectomy for the removal of an epidural abscess causing cauda equina and S1 nerve root compression. During the procedure, a dural puncture occurred, which was repaired using Duragen (collagen matrix) and Adherus (synthetic PEG hydrogel sealant). Postoperatively, the patient developed urinary retention and new bilateral posterior leg pain. An MRI on postoperative day four revealed a new peripherally enhancing dorsal intradural collection at the L2 level, causing significant thecal sac narrowing and compression of the cauda equina nerve roots, suggestive of migration of the hydrogel sealant used during surgery. Conservative management was adopted. **Results**: The patients symptoms gradually resolved. Follow-up imaging at five months showed resolution of the intradural collection, with residual intradural inflammatory changes and arachnoiditis. **Conclusions**: While PEG-based hydrogel sealants like Adherus are effective in preventing CSF leaks, they can, in rare instances, migrate and cause remote intradural collections with neurological symptoms. Surgeons should exercise meticulous application techniques, thoroughly document the use of sealants, and maintain vigilant postoperative monitoring to mitigate these risks.

## 1. Introduction

Laminectomy serves as a fundamental procedure in spine surgery, commonly performed to decompress the spinal cord and nerve roots [1]. While this intervention generally provides safe and effective treatment, there is risk for complications including dural tears, cerebrospinal fluid leaks, and postoperative hematoma formation [2]. Careful surgical techniques and the appropriate use of surgical sealants and hemostatic agents are necessary for the prevention and management of these complications [3].

Synthetic hydrogel sealants, such as polyethylene glycol (PEG)-based Adherus, have become increasingly utilized in neurosurgery to repair dural tears and prevent cerebrospinal fluid (CSF) leaks [4]. These products provide reliable dural closure through their hydrogel properties, making them valuable tools in the spine surgeon’s armamentarium. However, their use is not without risk, as these agents can potentially migrate from their initial application site before complete polymerization, leading to unexpected complications.

This case report documents an unusual complication where synthetic hydrogel sealant migration resulted in an intradural collection several levels above the surgical site. The case highlights a previously underreported risk of PEG-based dural sealants in spine surgery while emphasizing the critical importance of vigilant postoperative monitoring. Our findings suggest the need for careful consideration when using synthetic sealants, particularly in cases involving dural manipulation or repair where CSF dynamics may influence the behavior of these agents before they fully polymerize.

## 2. Case Presentation

**Figure 1:**
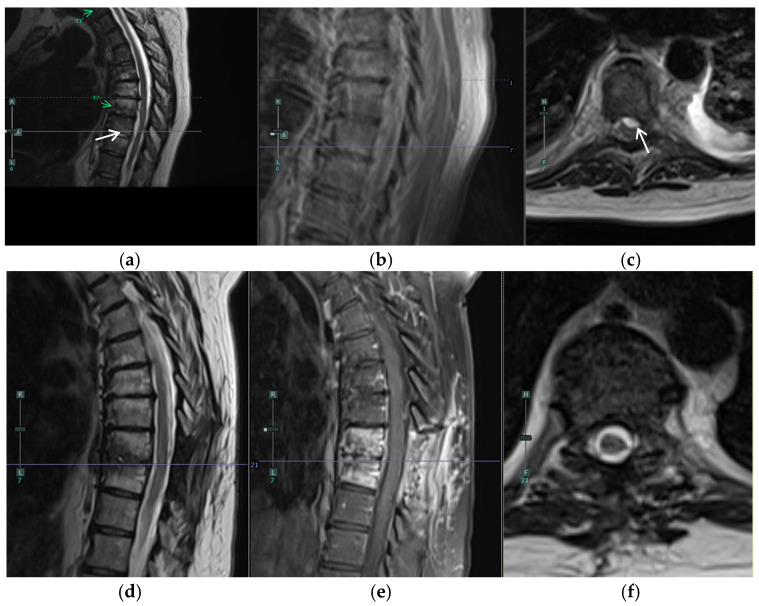
(**a**–**c**) MRI of the thoracic spine two months prior to operation prior to T8–T10 laminectomy for excision and drainage of a thoracic epidural abscess (white arrows) and left piriformis abscess aspiration: (**a**) Sagittal T2, (**b**) Sagittal T1 with contrast, and (**c**) Axial T2 at T8/T9. (**d**–**f**) Preoperative MRI of the thoracic spine, s/p original T8-T10 laminectomy, showing worsening diffuse lumbar facet infection and resolution of thoracic epidural abscess: (**d**) Sagittal T2, (**e**) Sagittal T1 with contrast, and (**f**) Axial T2 with contrast at T8/T9.

**Figure 2:**
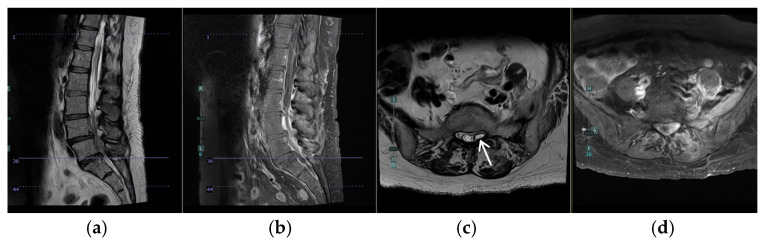
Preoperative MRI of the lumbar spine showing left-sided L5/S1 epidural abscess causing cauda equina and S1 root compression (white arrow). (**a**) Sagittal T2, (**b**) Sagittal T1 with contrast, (**c**) Axial T2 at S1, and (**d**) Axial T1 with contrast L5/S1.

A 57-year-old female with a past medical history of osteoarthritis and prediabetes presented with three days of worsening back and left leg pain. Her initial examination revealed significant tenderness in the lumbar region and decreased sensation in the left S1 distribution. She had preserved motor function but reported progressive difficulty with ambulation due to pain. Two months prior, she had undergone a T8–T10 laminectomy for excision and drainage of a thoracic epidural abscess and left piriformis abscess aspiration, followed by a six-week course of intravenous antibiotics (Figure 1a–c). Imaging at presentation demonstrated a resolving thoracic infection but revealed worsening diffuse lumbar facet infection and a left-sided L5/S1 epidural abscess causing cauda equina and S1 root compression (Figure 1d–f and Figure 2).

Given these findings, the patient underwent a minimally invasive L5-S1 laminectomy for abscess removal. Intraoperative findings included minimal purulent material but significant adhesive epidural phlegmon. A dural puncture occurred during the procedure, though no active CSF leak was observed. The durotomy was repaired using Duragen (collagen matrix), (Integra LifeSciences, Princeton, NJ, USA) and Adherus (synthetic PEG hydrogel sealant) (HyperBranch Medical Technology, Durham, NC, USA). Additional hemostatic agent Surgiflo (porcine gelatin matrix) (Ethicon, Raritan, NJ, USA) was applied, and Depomedrol was placed in the epidural space before closure. Intraoperative cultures subsequently grew methicillin-sensitive Staphylococcus aureus (MSSA).

**Figure 3:**
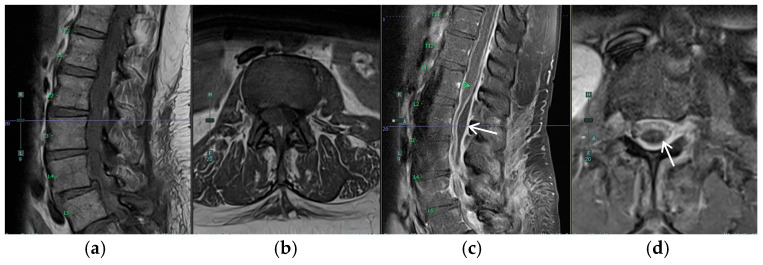
Postoperative MRI of the lumbar spine. The white arrows identify the new postoperative dorsal subdural collection, resulting in thecal sac narrowing and compression of the cauda equina nerve roots. (**a**) Sagittal T1, (**b**) Axial T1 at L3, (**c**) Sagittal T1 with contrast, green arrow: new peripherally enhancing dorsal subdural collection at the L2 level, and (**d**) Axial T1 with contrast at L3.

**Figure 4:**
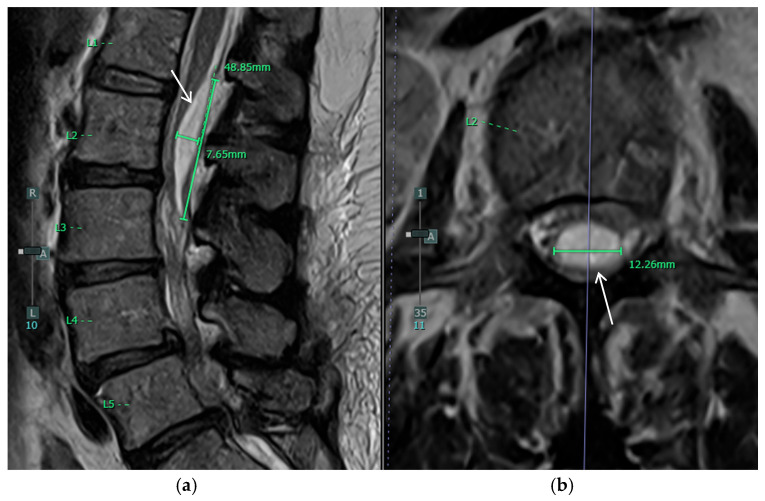
Postoperative MRI of the lumbar spine showing the new dorsal subdural fluid collection (white arrows). (**a**) Sagittal T2 and (**b**) Axial T2 at the level of L2.

The patient’s postoperative course was notable for the development of urinary retention and persistent back pain on postoperative day 2 (POD2). By POD3, she had developed new bilateral posterior leg pain with ambulation, in contrast to her preoperative unilateral left-sided symptoms. MRI on POD4 revealed an unexpected finding: a new peripherally enhancing dorsal subdural collection at the L2 level, significantly higher than the L5/S1 surgical site, resulting in moderate to severe thecal sac narrowing and compression of the cauda equina nerve roots (Figure 3 and Figure 4). The differential diagnosis for the intradural collection included several possibilities: migrated hydrogel sealant, epidural abscess, seroma, or acute hematoma. The imaging characteristics, particularly the peripheral enhancement pattern and the temporal relationship to sealant placement, along with the patient’s clinical course, were most consistent with migrated hydrogel sealant. Conservative management with close observation of symptom progression chosen given the patient’s stable neurological status and the known property of PEG-based hydrogels to degrade over time. The patient’s urinary retention resolved by POD6. Her bilateral leg pain showed improvement regarding POD9. Her symptom improvement further supported the diagnosis of migrated hydrogel sealant. She was discharged home with a six-week course of intravenous antibiotics.

**Figure 5:**
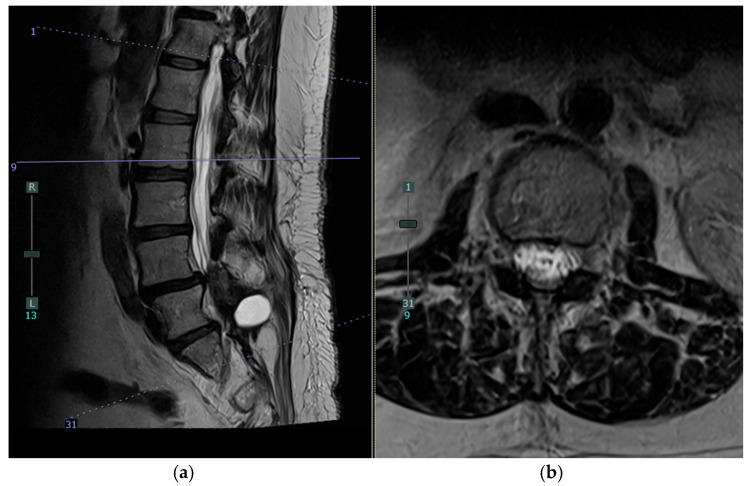
Postoperative MRI of the lumber spine five months after the operation showing resolution of the dorsal subdural collection. (**a**) Sagittal T2 and (**b**) Axial T2 at the level of L2.

At five-month follow-up, the patient’s leg pain had completely resolved, and she denied any weakness or numbness. Follow-up MRI at this time demonstrated absence of the previously described subdural fluid collection, resolution of the abscess, and improvement in phlegmon and septic arthritis, though residual intradural inflammatory changes and arachnoiditis remained (Figure 5). The resolution of the fluid collection on imaging and her resolved leg pain further supported the diagnosis of migrated hydrogel sealant.

## 3. Discussion

This case report highlights a rare but significant complication following spinal surgery, where migration of a synthetic polyethylene glycol (PEG) hydrogel dural sealant is suspected to have caused an intradural collection at a remote spinal level. Such occurrences are uncommon but underscore potential risks associated with the use of synthetic dural sealants, particularly in complex spinal surgeries involving altered anatomy or cerebrospinal fluid (CSF) dynamics.

### 3.1. Use of PEG-Based Hydrogel Sealants in Spinal Surgery

PEG-based hydrogel sealants, such as DuraSeal (Integra LifeSciences) and Adherus (previously by HyperBranch Medical Technology), are commonly used in spinal surgery to achieve watertight dural closure and prevent CSF leaks. These sealants polymerize upon application to form a hydrogel barrier that conforms to the dural surface and degrades over time through hydrolysis [5]. Large-scale clinical studies have demonstrated the efficacy of PEG-based hydrogel sealants, with randomized controlled trials showing high rates of watertight closure when used as an adjunct to sutured dural repair, though no method can guarantee complete prevention of CSF leaks [6]. Clinical studies have demonstrated their efficacy in reducing postoperative CSF leaks compared to conventional methods [7,8].

### 3.2. Mechanisms of Hydrogel Sealant Migration

Several factors may contribute to the migration of hydrogel sealants. The pulsatile nature of CSF flow can exert forces on the sealant before it fully polymerizes, potentially displacing it from the application site. PEG hydrogel sealants typically require about 60 s to achieve initial polymerization [9], during which time CSF pulsations may influence sealant positioning. In cases of durotomy or dural repair, altered CSF dynamics can exacerbate this effect.

The presence of significant scarring or disrupted tissue planes, as seen in patients with prior infections or surgeries, may create pathways for sealant migration. Scarring can disrupt normal anatomical barriers and facilitate the movement of materials within the spinal canal. Additionally, the application technique and volume of sealant used are critical factors; excessive application could in theory lead to mass effect or unintended spread of the materials, which is why unique formulations of these hydrogels for the spinal canal have been made [6,10]. Interactions with other surgical materials, such as hemostatic agents or dural substitutes, may alter the local environment and affect sealant behavior, although the specific mechanisms are not well understood.

### 3.3. Clinical Implications and Management

The development of an intradural collection at a spinal level remote from the surgical site poses significant diagnostic and therapeutic challenges. In patients presenting with neurological deficits or progressive symptoms, surgical intervention may be necessary to prevent permanent neurological damage. However, when the patient remains neurologically stable, a conservative management approach may be appropriate. The decision for conservative management in this case was based on several factors: the patient’s stable neurological status, gradual improvement in symptoms, and the known properties of PEG-based hydrogels to degrade over time. The risks of additional surgery were carefully weighed against the potential benefits, particularly given the patient’s improving clinical course.

PEG-based hydrogel sealants like Adherus are engineered to degrade gradually over several weeks to months through hydrolysis [9]. This property allows clinicians to consider close clinical and radiological monitoring as an alternative to immediate surgical removal. Regular imaging follow-up is essential to assess the degradation of the sealant and to monitor for any changes in the size or impact of the intradural collection. This conservative approach requires a multidisciplinary effort, involving neurosurgeons, radiologists, and neurologists, to ensure patient safety and optimal outcomes.

### 3.4. Prevention Strategies

Minimizing the risk of sealant migration necessitates strict adherence to recommended application techniques. Controlled application as well as utilization of correct formulations of the hydrogel is crucial; surgeons should use only the minimal effective volume of sealant to achieve dural closure, as overapplication, in theory, can increase the risk of migration and cause mass effect within the spinal canal [6,10]. Allowing sufficient time for the sealant to polymerize before closing the surgical site is equally important. PEG hydrogels typically require approximately 60 s to achieve initial polymerization [9], and premature closure may predispose the sealant to displacement by CSF pulsations.

Patient selection and preoperative planning are also vital. Surgeons should thoroughly evaluate the patient’s anatomy for any alterations due to previous surgeries, infections, or scarring that might increase the risk of sealant migration. In such cases, surgical techniques may need to be adjusted to account for disrupted CSF dynamics. Additionally, caution should be exercised when using multiple adjunctive materials, e.g., hemostatic agents or dural substitutes. Interactions between these materials and the hydrogel sealant could affect the local microenvironment and potentially alter the sealant’s behavior, although the specific mechanisms are not yet fully understood.

### 3.5. Lessons Learned and Recommendations

This case highlights several critical considerations for spinal surgery involving synthetic dural sealants. Meticulous application of the sealant according to the manufacturer’s instructions is essential to minimize complications. Precise placement and appropriate volume usage can prevent overapplication and reduce the risk of migration. Thorough documentation of the type, volume, and site of sealant application is important for postoperative assessment and can aid in managing any complications that may arise.

Awareness of the potential for sealant migration is crucial, particularly in patients with altered anatomy or disrupted CSF dynamics due to prior infections or surgeries. Preoperative planning should incorporate an assessment of these risk factors, and surgical strategies should be adapted accordingly. Vigilant postoperative monitoring is imperative for early recognition of complications and prompt imaging should be considered in patients presenting with new or unexplained symptoms.

Finally, there is a need for further research to better understand the behavior of synthetic dural sealants within the intradural space. Studies aimed at optimizing the viscosity and adherence properties of these sealants could reduce the risk of migration and enhance their safety profiles. Investigations into the interactions between hydrogel sealants and other surgical materials, as well as the influence of altered CSF dynamics, will be valuable in developing improved formulations.

## 4. Conclusions

While synthetic PEG hydrogel sealants like Adherus are effective in preventing cerebrospinal fluid leaks in spinal surgery, they can, in rare cases, migrate and cause complications such as remote intradural collections. This case underscores the importance of meticulous application techniques and vigilant postoperative monitoring to mitigate such risks. Awareness of potential complications and adherence to best practices are essential to improve patient outcomes in spinal surgery.

## Data Availability

The data presented in this study are available on request from the corresponding author due to the Health Insurance Portability and Accountability Act (HIPAA).

## Abbreviations

The following abbreviations are used in this manuscript:

PEG	Polyethylene glycol
CSF	Cerebrospinal fluid
MSSA	Methicillin-sensitive Staphylococcus aureus
POD	Postoperative day
